# Succinylcholine versus rocuronium for rapid sequence intubation in intensive care: a prospective, randomized controlled trial

**DOI:** 10.1186/cc10367

**Published:** 2011-08-16

**Authors:** Stephan C Marsch, Luzius Steiner, Evelyne Bucher, Hans Pargger, Martin Schumann, Timothy Aebi, Patrick R Hunziker, Martin Siegemund

**Affiliations:** 1 Department of Medical Intensive Care, University Hospital Basel, University of Basel, Petersgraben 4, Basel 4031, Switzerland; 2 Department of Surgical Intensive Care, University Hospital Basel, University of Basel, Petersgraben 4, Basel 4031, Switzerland

## Abstract

**Introduction:**

Succinylcholine and rocuronium are widely used to facilitate rapid sequence induction (RSI) intubation in intensive care. Concerns relate to the side effects of succinylcholine and to slower onset and inferior intubation conditions associated with rocuronium. So far, succinylcholine and rocuronium have not been compared in an adequately powered randomized trial in intensive care. Accordingly, the aim of the present study was to compare the incidence of hypoxemia after rocuronium or succinylcholine in critically ill patients requiring an emergent RSI.

**Methods:**

This was a prospective randomized controlled single-blind trial conducted from 2006 to 2010 at the University Hospital of Basel. Participants were 401 critically ill patients requiring emergent RSI. Patients were randomized to receive 1 mg/kg succinylcholine or 0.6 mg/kg rocuronium for neuromuscular blockade. The primary outcome was the incidence of oxygen desaturations defined as a decrease in oxygen saturation ≥ 5%, assessed by continuous pulse oxymetry, at any time between the start of the induction sequence and two minutes after the completion of the intubation. A severe oxygen desaturation was defined as a decrease in oxygen saturation ≥ 5% leading to a saturation value of ≤ 80%.

**Results:**

There was no difference between succinylcholine and rocuronium regarding oxygen desaturations (succinylcholine 73/196; rocuronium 66/195; *P *= 0.67); severe oxygen desaturations (succinylcholine 20/196; rocuronium 20/195; *P *= 1.0); and extent of oxygen desaturations (succinylcholine -14 ± 12%; rocuronium -16 ± 13%; *P *= 0.77). The duration of the intubation sequence was shorter after succinycholine than after rocuronium (81 ± 38 sec versus 95 ± 48 sec; *P *= 0.002). Intubation conditions (succinylcholine 8.3 ± 0.8; rocuronium 8.2 ± 0.9; *P *= 0.7) and failed first intubation attempts (succinylcholine 32/200; rocuronium 36/201; *P *= 1.0) did not differ between the groups.

**Conclusions:**

In critically ill patients undergoing emergent RSI, incidence and severity of oxygen desaturations, the quality of intubation conditions, and incidence of failed intubation attempts did not differ between succinylcholine and rocuronium.

**Trial Registration:**

ClinicalTrials.gov, number NCT00355368.

## Introduction

Endotracheal intubation in critically ill patients is a high risk procedure containing the danger of hypoxia and cardiovascular collapse. The method of choice for emergency intubation in the intensive care unit (ICU) is a rapid sequence induction (RSI). Because of its fast onset, succinylcholine is the most commonly used neuromuscular blocking drug in RSI. Due to its depolarizing mechanism of action resulting in an increase in extracellular potassium, succinylcholine is contraindicated in a number of circumstances and diseases frequently present in critically ill patients [1]. Rocuronium has the most rapid onset of the currently available non-depolarizing neuromuscular blocking drugs. As the only contraindication to rocuronium is the very rare occasion of allergy, this agent is regarded as an attractive alternative to succinylcholine [2,3]. Assessing current clinical practice by analyzing large contemporary trials reveals a wide variation in the use of rocuronium or succinylcholine for RSI in the ICU [4-8]. However, neither agent has been so far tested against each other or any other neuromuscular blocking agent in an adequately powered randomized trial in critically ill patients.

Compared to succinylcholine, rocuronium is associated with less optimal intubation conditions [3,9] and a longer intubation sequence [9] in the operating theatre. As the most frequent complication of RSI in the ICU is severe hypoxemia [4,7] the combination of less optimal intubation conditions and longer intubation sequence may be of relevance. Given the low rate of hypoxemia during RSI in the operating theatre, available studies are underpowered for this outcome. The aim of this prospective randomized controlled trial was, therefore, to compare the incidence of hypoxemia after rocuronium or succinylcholine in critically ill patients requiring an emergent RSI.

## Material and methods

### Design

This is a prospective randomized controlled single-blind trial. The trial is registered, ClinicalTrials.gov, number NCT00355368.

### Setting

The trial took place in the medical and surgical ICUs of the University Hospital of Basel, a tertiary care center.

### Patients

All adult (age ≥ 18 years) patients requiring emergent endotracheal intubation with a RSI were eligible. The indication for intubation was made by the staff physician in charge of the patient's care. Patients could be included only once in the trial. Exclusion criteria were contraindications against succinylcholine (that is, hyperkalemia, neuromuscular diseases, denervation of muscles, tetraplegia, long-term immobilization, extensive muscle trauma, burns, familial history of malignant hyperthermia), allergy to rocuronium, pregnancy, known or anticipated difficult intubation warranting awake fiberoptic intubation, and absence of a qualified study physician to perform the intubation. Patients excluded from the study because of the absence of a qualified study physician and those erroneously included more than once were included in a registry. Acute Physiology And Chronic Health Evaluation II (APACHE II) scores [10] were calculated for each patient based on the 24 hours preceding intubation.

The study was conducted in compliance with the Helsinki Declaration and was approved by the regional Ethics Committee (Ethikkommission beider Basel, Basel, Switzerland). Because critically ill patients requiring an emergent intubation are typically unable to give informed consent, the regional Ethics Committee granted a waiver of consent prior to the intubation. Instead, investigators were obliged to inform relatives and obtain written informed consent from patients as soon as feasible.

### Intervention

Stratified randomization by gender was used to ensure a similar distribution of gender in both groups. Using sealed envelopes, patients were randomly allocated by the study physician to receive either 0.6 mg/kg rocuronium (Esmeron^®^, Organon, Pfäffikon, Switzerland) or 1.0 mg/kg succinylcholine (Lystenon^®^, Nycomed, Opfikon, Switzerland) intravenously as neuromuscular blocking drug.

Preparations for intubation followed a checklist (Additional file 1). Intubations were performed or supervised by a study physician, defined as a physician with dual training in anaesthesia (board certified) and critical care (board certified or in the last year of training). The pre-intubation management including the position of the head of the bed (horizontal or elevated), the application of cricoid pressure [11], and the management of difficulties and complications, if any, was at the discretion of the study physicians. The protocol encouraged, time permitting, cardiovascular optimization prior to intubation using fluids and catecholamine infusion. In patients undergoing non-invasive ventilation, the protocol encouraged continued non-invasive ventilation with 100% oxygen as the means of pre-oxygenation. All other patients were pre-oxygenated using a bag-mask device with high flow oxygen. Patients receiving light sedation to tolerate non-invasive ventilation continued to do so until the beginning of the induction sequence.

A total of 1 μg/kg intravenous fentanyl was administered at the beginning of the three-minute pre-oxygenation period. Thereafter, an intravenous induction agent was administered: etomidate 0.2 mg/kg in patients with a mean arterial pressure < 80 mmHg and/or a catecholamine infusion; propofol 1 mg/kg in all other patients. The neuromuscular blocking drug was injected as soon as the injection of the induction agent was completed. Laryngoscopy was started after the cessation of fasciculations in the lower extremities [12], if any, or after 45 sec (anticipated time of intubation 60 sec after the injection of the neuromuscular blocking drug), whichever was earlier. Intubations were performed using a Macintosh size 3 blade and a tracheal tube (Mallinckrodt Hi-Contour, Mallinckrodt, Ireland) with an internal diameter of 8.0 cm. The timing of events was performed using a stopwatch.

### Outcome measures

The primary outcome was the incidence of oxygen desaturations defined as a decrease in oxygen saturation ≥ 5%, assessed by continuous pulse oxymetry, at any time between the start of the induction sequence and two minutes after the completion of the intubation. A severe oxygen desaturation was defined as a decrease in oxygen saturation of ≥ 5% leading to a saturation value of ≤ 80%.

Secondary outcomes were 1) the duration of the intubation sequence, defined as the time interval between the injection of the induction agent and the first appearance of end-tidal carbon dioxide on the screen of the monitor; 2) the incidence of failed first intubation attempts; 3) numerical [2] and qualitative [13] intubation conditions as rated by the intubating study physician using a scoring system proposed for good clinical research practice in studies of neuromuscular blocking drugs [14] (Table 1); and 4) haemodynamic consequences of intubation between the start of the induction sequence and five minutes after the completion of the intubation.

**Table 1:** Scoring system for intubation conditions

	Score 3	Score 2	Score 1
*Laryngoscopy*			
Jaw relaxation	Relaxed	Acceptable relaxation	Poor relaxation
Resistance to blade	None	Slight resistance	Active resistance
*Vocal cords*			
Position	Abducted	Intermediate	Closed
Movement	None	Moving	Closing
*Intubation response*			
Limb movement	None	Slight	Vigorous
Coughing	None	Diaphragmatic	Severe coughing or bucking

Table 1 shows the scoring system used to derive both a numerical and qualitative intubation score. The factors laryngoscopy, vocal cords, and response to intubation are individually rated with a score from 1 (worst) to 3 (best). The assignment of a score for each of the three factors is based on the lower rating of two parameters. For example, the combination of the parameters "no limb movement" and "no coughing" results in a score of 3 for the factor response to intubation, while the combination of the parameters "no limb movement" and "severe coughing" results in a score of 1.

The numerical intubation score was obtained by summing up the scores assigned to the factors: laryngoscopy, vocal cords, and response to intubation. The maximum score is thus 9, while the minimum score is 3.

The qualitative intubation scores were defined as follows: (a) Excellent intubation conditions: all three factors were rated with a score of 3. (b) Good intubation conditions: all three factors were rated either with a score of 3 or 2. (c) Poor intubation conditions: the presence of one factor rated with a score of 1.

Excellent and good intubation conditions are considered clinically acceptable while poor intubation conditions are considered clinically not acceptable [14].

### Statistical analysis

Data, presented as means ± SD unless otherwise stated, were analyzed using SPSS 15.0 for Windows (SPPS Inc., Chicago, IL, USA). Two-way ANOVA, unpaired Student's *t*-test, Mann-Whitney test, Fisher's exact test and the logrank test were applied as appropriate. Based on data from the literature we estimated that approximately 250 patients were required for each study group to detect a difference of 20% in the primary outcome, incidence of oxygen desaturation ≥ 5%, with a power of 0.9 and a two-sided α of 0.05. A planned interim power analysis, performed with the results of the first 100 patients of the succinylcholine group, revealed that, in fact, approximately 200 patients were required for each study group. To account for protocol violations related to an emergent procedure we planned to enroll 210 patients per group.

## Results

**Figure 1:**
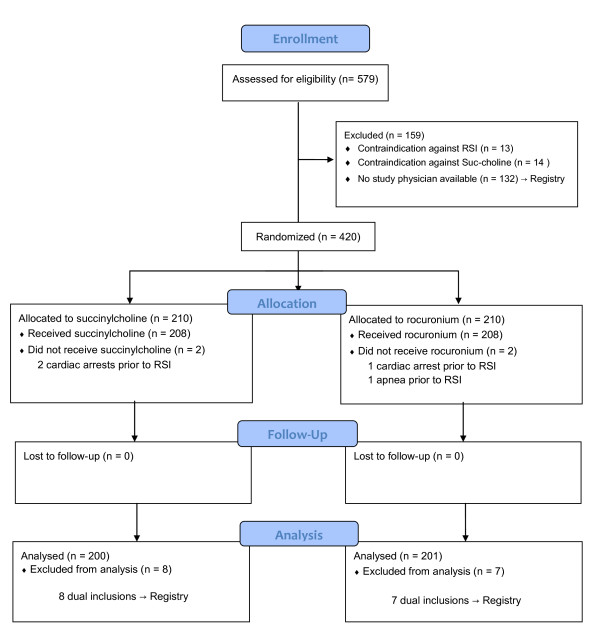
**Patients' flow diagram reported in CONSORT style**. RSI, rapid sequence induction (intubation).

The study started in August 2006 and ended with the inclusion of the 420^th ^patient in June 2010. Figure 1 shows the patients' flow. Of the 401 intubations 333 (83%) were accomplished on the first, 57 (14.2%) on the second, 9 (2.2%) on the third, and 2 (0.5%) on the fourth attempt. No periprocedural death occurred. Table 2 shows the indications for the intubations performed. Demographics of the patients included in the study and the registry are shown in Table 3.

**Table 2:** Indications for emergent intubations in 401 critically ill patients

	ICD-10	N
**Respiratory indications for intubation (*n *= 264)**		
Respiratory failure due to sepsis	A41	53
Pneumonia, hospital-acquired	J13 to J15	53
Pneumonia, community-acquired	J13 to J15	51
Respiratory failure ≤ 24 h after extubation	J95.8	36
Exacerbation of COPD (chronic obstructive pulmonary disease)	J44.0	22
Pulmonary oedema	J81	10
ARDS (adult respiratory distress syndrome)	J80	7
Aspiration of blood (*n *= 3) or gastric contents (*n *= 4)	W78	7
Thoracic trauma	S22	5
Pulmonary haemorrhage	R04.8	4
Respiratory failure ≤ 24 h after self-extubation	J96.0	4
Respiratory failure due to massive pulmonary secretion	J96.0	4
Respiratory failure due to dislocation of a tracheal cannula	J96.0	3
Pancreatitis	K85	2
Pulmonary oedema due to inhalational trauma	J68.1	1
Malignant neoplasm of larynx	C32	1
Angioneurotic oedema of the tongue	T78.3	1
**Neurologic indications for intubation (*n *= 92)**		
Epilepsy and Status epilepticus with GCS ≤ 6	G40, G41	33
Hepatic coma with GCS ≤ 6	K72.0	9
Poisoning with GCS ≤ 6	T40, T42	9
Intracerebral haemorrhage with GCS ≤ 6	I61	7
Guillain-Barré syndrome	G61.0	6
Cerebral infarction with GCS ≤ 6	I63	5
Coma of unknown origin with GCS ≤ 6	G93.9	5
Subarachnoidal haemorrhage with GCS ≤ 6	I62	4
Delirium	F05	4
Myasthenia gravis	G70.0	3
Cerebral venous throbosis with GCS ≤ 6	I63.6	2
Diabetic coma with GCS ≤ 6	E10.0	1
Encephalitis with GCS ≤ 6	G04	1
Meningitis with GCS ≤ 6	G00	1
Traumatic cerebral oedema with GCS ≤ 6	S06.1	1
Thrombotic thrombocytopenic purpura with GCS ≤ 6	M31.1	1
**Shock as indication for intubation (*n *= 45)**		
Cardiogenic shock due to acute myocardial infarction	I23.8	18
Cardiogenic shock due to acute non-ischaemic heart disease	R57.0	7
Septic shock	A41.9	13
Haemorrhagic shock	R57.1	7

ICD categories relate to the current (2007) version of the WHO (World Health Organisation).

**Table 3:** Demographics

	Succinylcholine (*N *= 200)	Rocuronium (*N *= 201)	Registry (*N *= 147)
Age (years)	60 ± 16	63 ± 14	59 ± 16
Sex (m:f)	114:86	112:89	94:54
Height (cm)	170 ± 8	170 ± 9	171 ± 10
Weight (kg)	73 ± 15	74 ± 19	74 ± 11
Apache II Score	21 ± 7	22 ± 7	21 ± 6
Underlying COPD	32 (16%)	30 (15%)	27 (13%)
28-day mortality	73 (37%)	82 (41%)	53 (36%)
Indication for intubation			
Respiratory failure	134 (67%)	130 (65%)	91 (62%)
Neurology	42 (21%)	50 (25%)	41 (28%)
Shock	24 (12%)	21 (10%)	15 (10%)
Non-invasive ventilation prior to intubation	93 (47%)	85 (43%)	62 (42%)
Induction agent			
Propofol	101 (50%)	94 (47%)	47 (32%)
Etomidate	99 (50%)	107 (53%)	67 (46%)
Other			33 (22%)
Neuromuscular blocking agent			
Succinylcholine			78 (53%)
Rocuronium			44 (30%)
Other			25 (17%)

The registry includes patients who were intubated while the trial was recruiting but were not included in the trial due to the unavailability of a study physician (*n *= 132) or had to be excluded from the trial due to dual inclusion (*n *= 15).

The observed 28-day mortality corresponds to the expected mortality as APACHE II scores of 21 and 22 predict a mortality of approximately 39% and 42% respectively [10]. COPD, chronic obstructive pulmonary disease.

### Primary outcome

**Figure 2:**
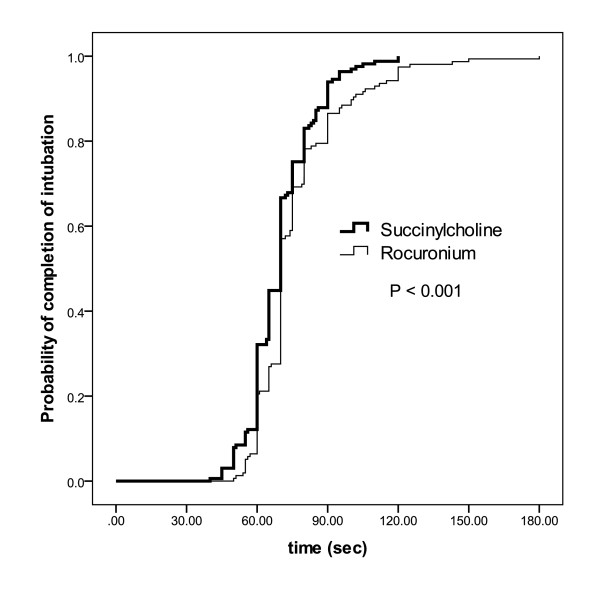
**Intubating times**. Kaplan-Meyer curve of the probability of the completion of the endotracheal intubation sequence including succinylcholine or rocuronium in patients successfully intubated in the first attempt. The x-axis denotes the time interval after the beginning of the injection of the induction drug. The intubation sequence was defined to be completed upon the first appearance of end-tidal carbon dioxide after intubation.

Due to a severe shock state, pulse oxymetry could not measure oxygen saturation in four patients of the succinylcholine group and in six patients of the rocuronium group. In the remaining patients, there was no significant difference in the incidence of oxygen desaturations (succinylcholine 73/196 = 37%; rocuronium 66/195 = 34%; *P *= 0.67) and severe oxygen desaturations (succinylcholine 20/196 = 10%; rocuronium 20/195 = 10%; *P *= 1.0) between the groups. In those patients exhibiting a decrease in oxygen saturation ≥ 5%, there was no difference between the groups with regard to the extent of the decrease (succinylcholine -14 ± 12%; rocuronium -16 ± 13%; *P *= 0.77). Figure 2 displays the course of the oxygen saturation over the intubation period.

### Secondary outcomes

**Figure 3:**
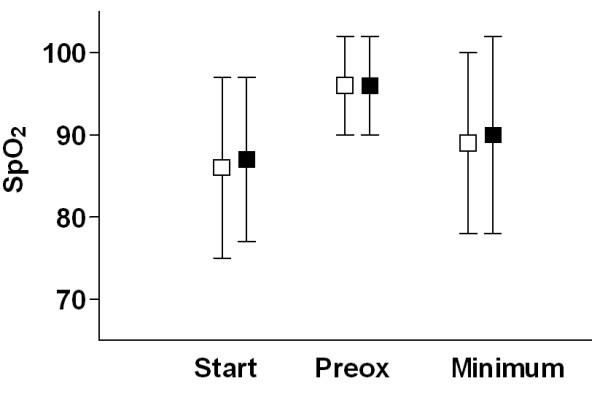
**Oxygen saturations**. Oxygen saturation (SpO_2_) obtained by pulsoxymetry at the beginning of the pre-oxygenation period (Start), after completion of pre-oxygenation (Preox), and minimum value at any time between the start of the induction sequence and two minutes after the completion of the intubation respectively. Data are the means ± SD. There was no statistically significant difference between succinylcholine and rocuronium.

**Figure 4:**
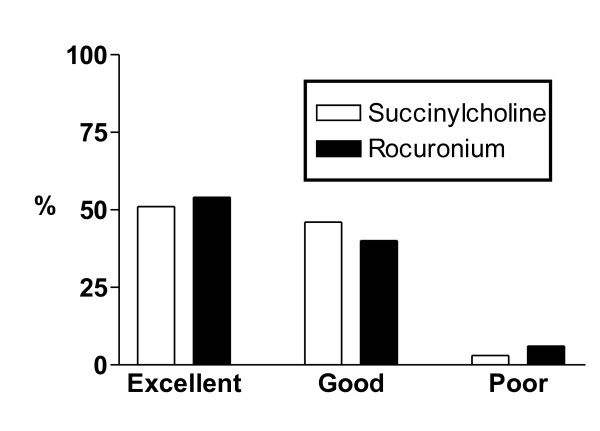
**Intubating conditions**. Intubation conditions during rapid sequence induction intubation with succinylcholine or rocuronium. The scoring system is explained in Table 1. There were no significant differences between the two neuromuscular blocking drugs.

More than one intubation attempt was required in 32/200 patients under succinylcholine and in 36/201 patients under rocuronium (*P *= 0.4; Table 3). In 77/200 (39%) patients of the succinylcholine group fasciculations were not visible or had not ceased 45 sec after the injection of the drug. Figure 3 depicts the duration of the intubation sequence which was significantly shorter (*P *= 0.002) in the succinylcholine group (81 ± 38 sec) than in the rocuronium group (95 ± 48 sec). Intubation was not completed within 90 sec in 50/200 patients under succinylcholine and 67/201 patients under rocuronium (*P *= 0.048). Qualitative scores of intubation conditions are shown in Figure 4. The numerical sub-scores for ease of laryngoscopy (succinylcholine 2.75 ± 0.45; rocuronium 2.75 ± 0.46; *P *= 0.84) and conditions of the vocal cords (succinylcholine 2.61 ± 0.52; rocuronium 2.67 ± 0.56; *P *= 0.32) did not differ between the groups while there was a small, but significant difference in the sub-score of the response to intubation (succinylcholine 2.97 ± 0.20; rocuronium 2.86 ± 0.36; *P *= 0.001). The overall numerical score for intubation conditions (succinylcholine 8.3 ± 0.8; rocuronium 8.2 ± 0.9; *P *= 0.7) did not differ between the groups. There was no difference between the groups in the incidence of hemodynamic consequences of intubation (Table 4).

**Table 4:** Incidence of complications other than oxygen desaturation of emergent intubations in 401 critically ill patients

	Succinylcholine (*n *= 200)	Rocuronium (*n *= 201)
Failed first intubation attempt	32 (16%)	36 (18%)
Anatomical difficult airway*	10 (5%)	12 (6%)
Difficult laryngoscopy^§^	7 (3.5%)	5 (2.5%)
Oesophageal intubation	2 (1%)	2 (1%)
Equipment problems	1 (0.5%)	3 (1.5%)
Aspiration^†^	4 (2%)	3 (1.5%)
Need of a vasopressor after intubation^¶^	84 (42%)	90 (45%)
Cardiac arrest	6 (3%)	4 (2%)
Ventricular fibrillation	2 (1%)	1 (0.5%)
Pulsless electrical activity	4 (2%)	3 (1.5%)
Asystole	0	0
Death	0	0

* An anatomically difficult airway was defined as more than two attempts or a successful second attempt with the help of additional equipment (for example, a different blade) or a modified technique (for example, preforming a stylet).

^§ ^Difficult laryngoscopy was defined as impaired vision of laryngeal structures due to non-anatomical reasons (for example, blood or secretion).

† Blood, gastric content, or foreign body visible below the vocal cords during laryngoscopy

¶ Need of vasopressor was defined as an intravenous bolus of a vasopressor or an increase in infusion rate of a running catecholamine infusion.

## Discussion

During the course of an emergent intubation the incidence and severity of oxygen desaturations did not differ between critically ill patients undergoing RSI with succinylcholine and those undergoing RSI with rocuronium as the neuromuscular blocking agent. The mean intubation sequence was 14 sec shorter after succinylcholine than after rocuronium. Succinylcholine and rocuronium resulted in similar intubation conditions and a similar incidence of intubation related complications.

A recent Cochrane Review demonstrated that for a RSI succinylcholine created better intubation conditions than rocuronium [3]. However, all patients included in the Cochrane Review were intubated in the operating theatre. Conditions in intensive care and in the emergency department may differ in several important aspects from those in the operating theatre (for example, severity of patients' illness, limited possibility to perform a pre-intubation airway assessment, less ideal ergonomic conditions) so that findings from one setting are not necessarily applicable to the other. Particularly, the incidence of relevant intubation-related complications is very small in the operating theatre so that available studies are underpowered for these outcomes. In keeping with previous work, the present study demonstrates that emergent intubations in intensive care are associated with a high rate of immediate and potentially life-threatening complications like oxygen desaturations and failed intubation attempts [4,5,15].

In contrast to the majority of studies performed in the operating theatre [3], the present study demonstrated no difference in intubation conditions between succinylcholine and rocuronium. In emergency department RSI, Lauren *et al*. reported that succinylcholine resulted in less body movements as a reaction to intubation than rocuronium, while there was no difference between the two drugs with regard to the degree of vocal cord movements [16]. In emergent RSI in the operating theatre, Sluga *et al*. reported that the difference in the overall intubation scores between succinylcholine and rocuronium was entirely due to a difference in the sub-score assessing the intubation response, that is, limb movements and/or coughing, while there was no difference in the remaining sub-scores assessing the ease of laryngoscopy or the conditions of the vocal cords [9]. The previous finding of similar sub-scores for laryngoscopy and vocal cords is confirmed by our results. As limb movements and/or coughing were only rarely observed in the present study, sub-scores for the intubation response were very high in both groups and did not result in a difference in the overall score for intubation conditions. This absent response to intubation in the majority of critically ill patients is most likely due to the severity of the underlying illness. Based on the present results and previous findings [9,16] we propose that the difference reported for intubation conditions between succinylcholine and rocuronium [3] results entirely from a difference in the response to intubation, an event occurring after the completion of the intubation with marginal relevance for patients' safety. By contrast, succinylcholine and rocuronium do not differ in two aspects of intubation conditions highly relevant for patients' safety - the ease of laryngoscopy and the conditions of the vocal cords.

During emergent intubations in the operating theatre, succinylcholine allowed for a 35 sec earlier completion of the intubation sequence than rocuronium [9]. In the present study involving critically ill patients, this favorable effect of succinylcholine was reduced to 14 sec, that is, to approximately 15% of the total length of the intubation sequence with rocuronium. In almost 40% of our patients visible fasciculations had not occurred or had not ceased within 45 sec after the injection of succinylcholine, and, according to study protocol, laryngoscopy was started in these patients at the same time as in the rocuronium group. To the best of our knowledge, a delayed onset of succinylcholine-induced fasciculations in critically ill patients has not been reported so far. As severe illness may have a profound effect on the neuromuscular system [17] it is tempting to speculate that the severity of underlying illness is responsible for the difference in the onset of fasciculations and, hence, the reduced advantage of succinylcholine with regard to rapid completion of the intubation sequence between patients in the operating theatre and patients in intensive care.

A limitation of this trial is the lack of a double-blind design. However, masking the effects of drugs like succinylcholine that have visible effects (fasciculations) is inherently difficult. A RSI in critically ill patients is a high-risk procedure requiring the full attention of an appropriately trained physician. Since in our settings the simultaneous achievement of perfect blinding and optimal patient safety was not feasible, we opted for a single-blind study design. Using experienced operators and a checklist (containing all relevant aspects recently found to decrease complications related to intubation in intensive care [5]), every effort was made to protect the patients' safety. Thus, our results are not necessarily generalisible to settings with less stringent procedural guidelines and/or less experienced operators. Our findings were obtained with an induction sequence of fentanyl and propofol or etomidate and cannot be extrapolated to other drugs and/or doses.

The dose of rocuronium was chosen based on the best evidence available at the time of the design of the trial, that is, the Cochrane Review of 2003 [2]: For the outcome acceptable versus suboptimal intubating conditions, this review revealed no significant difference between succinylcholine and rocuronium at a dose of 0.6 to 0.7 mg/kg. Moreover, a sensitivity analysis within the propofol induction group (*n *= 640) for dose of rocuronium used (0.6 to 0.7 mg/kg versus 0.9 to 1.0 mg/kg) demonstrated that dose did not alter intubating conditions. Weighing the risk of prolonged neuromuscular blockade associated with higher doses of rocuronium against the lack of convincing evidence of better intubating conditions, we chose the smallest dose proven to be effective, that is, 0.6 mg/kg. However, the 2008 update of the Cochrane Review demonstrated an advantage of succinylcholine against lower (0.6 to 0.7 mg/kg), but not against higher (> 0.9 mg/kg), doses of rocuronium for the outcome acceptable versus suboptimal intubating conditions [3]. As the difference is small (risk ratio 0.95, 95% confidence intervals 0.90 to 0.99) and, as extensively discussed above, appeared to be mainly caused by the response to intubation [9], we decided not to modify the protocol of our ongoing trial. Our trial demonstrates identical intubating conditions with 1 mg/kg succinylcholine and 0.6 mg/kg rocuronium. Thus, in critically ill patients there is not an advantage in choosing higher doses of rocuronium than 0.6 mg/kg.

## Conclusions

The adverse effect profile of succinylcholine is of concern and some authors even considered its use in the ICU as obsolete [1]. Optimal intubation conditions [2,3], a short intubation sequence [9], and the return of spontaneous respiratory activity within 5 to 10 minutes are the main arguments of the proponents of the use of succinylcholine in the ICU [18,19]. The present study is the first formal comparison of succinylcholine and rocuronium in an adequately powered randomized controlled trial in critically ill patients. Our results demonstrate that in emergent intubations in intensive care 1) the incidence and severity of hypoxaemia does not differ between rocuronium and succinylcholine; 2) intubating conditions do not differ between rocuronium and succinylcholine; 3) the advantage of succinylcholine with regard to the length of the intubation sequence is markedly reduced to a difference of questionable clinical significance; and 4) the incidence of failed intubation attempts does not differ between rocuronium and succinylcholine. Lee *et al*. reported that the reversal of profound rocuronium-induced neuromuscular block with sugammadex was significantly faster than spontaneous recovery from succinylcholine [20]. Taking together its favourable safety profile, the availability of a rapid-acting antagonist, and our present results, rocuronium appears to be more than a suitable alternative to succinylcholine for emergent RSI intubations in acutely ill patients.

## Key messages

■ This is the first formal comparison of succinylcholine and rocuronium for use in emergent rapid sequence induction intubation in intensive care in an adequately powered randomized controlled trial.

■ In critically ill patients, undergoing an emergent rapid sequence induction intubation, the incidence and severity of oxygen desaturations, the quality of intubation conditions, and the incidence of failed intubation attempts did not differ between succinylcholine and rocuronium.

## Abbreviations

APACHE II: Acute Physiology And Chronic Health Evaluation; COPD,: chronic obstructive pulmonary disease; ICU: intensive care unit; RSI: rapid sequence induction (intubation): SpO_2: _oxygen saturation.

## Competing interests

The authors declare that they have no competing interests.

## Authors' contributions

SM had full access to all of the data in the study and takes responsibility for the integrity of the data, and the accuracy of the data analysis. SM was a principal investigator, and conceived and designed the trial and developed the protocol and the statistical analysis plan, participated in the recruitment of patients and performed or supervised intubations and drafted the manuscript. HP and MSie were principal investigators, conceived and designed the trial and developed the protocol and the statistical analysis plan, participated in the recruitment of patients and performed or supervised intubations. PH conceived and designed the trial and developed the protocol and the statistical analysis plan and reviewed the safety data. LS, EB, MSch and TA participated in the recruitment of patients and performed or supervised intubations. All authors read and approved the final manuscript.

## Supplementary Material

Additional file 1 **Checklist of preparations to be performed prior to RSI**. Click here for file
