# Supplementary material for: Succinylcholine versus rocuronium for rapid sequence intubation in intensive care: a prospective, randomized controlled trial
Source: Crit Care. 2011 Aug 16;15(4):R199. doi: 10.1186/cc10367 (PMC3387641; doi:10.1186/cc10367)
Supplement: Additional file 1 — Checklist of preparations to be performed prior to RSI. [file cc10367-S1.DOC]

# Additional file 1: Checklist Intubation

1. Checklist for the nurse in charge of the patient

*Preparation of equipment*

CO_2_-modul present and checked

Patient equipped with pulse-oxymeter

Box with regular intubation equipment present

Box "difficult intubation" present

Laryngoscope checked (light works)

Reserve laryngoscope checked

Magill forceps present (leave in package)

Tube (ID 8.0) present, cuff ckecked

Reserve tube present (leave in package)

Suction present and checked

Facemask and bag present and checked

Stethoscope present

Material for tube-fixation present

*Preparation of drugs*

*The following drugs should be immediately available at bedside:*

Etomidate (2 mg/ml): 3 ampoules (10 ml)

Fentanyl (50 µg/ml): 2 ampoules (2 ml)

Midazolam (1 mg/ml): 1 ampoule (5ml)

Succinylcholine (50 mg/ml): 1 ampoule (2ml)

Rocuronium (10 mg/ml): 1 ampoule (10ml)

Atropine (0,5 mg/ml): 3 ampoules (1ml)

Ephedrine (5 mg/ml): 1 ampoule (10 ml)

*Further preparations*

Free infusion line, including 3-way stopcock and

running infusion, available for injection of drugs

Switch monitor to make pulse-oximetry signal audible

Presence of a 2nd nurse at bedside

2. Checklist for the physician performing the intubation

*You are responsible for*

The complete management of the intubation

The selection of drugs

Task distribution within your team

Immediate confirmation of the correct position of the tube

Initial setup of the ventilator

*If time permits consider*

Stabilise haemodynamics with fluids and catecholamine infusion

Use non-invasive ventilation (NIV) for pre-oxygenation. If patient is already on NIV, continue NIV.
